# Practice in Nursery Weed Control—Review and Meta-Analysis

**DOI:** 10.3389/fpls.2021.807736

**Published:** 2022-02-02

**Authors:** Ping Yu, Stephen Christopher Marble

**Affiliations:** Department of Environmental Horticulture, Mid-Florida Research and Education Center, University of Florida Institute of Food and Agricultural Sciences, Apopka, FL, United States

**Keywords:** container, weed efficacy, herbicide, non-chemical, integrated control, citespace

## Abstract

Weeds, as one of the biggest challenges in the nursery industry, have been controlled by various methods, such as chemical and non-chemical practices. Although these practices have been widely established and tested to control weeds, there is no systematic or meta-analysis review to provide quantitative weed control efficacy information of these practices. To provide a systematic understanding of weed control practices in nursery production, a visualization research trend, a systematic review, and a meta-analysis were conducted. A total of 267 relevant studies were included for the research trend and 83 were included in the meta-analysis. The results in this study showed that interests in nursery weed control have switched dramatically in the past 2–3 decades (1995–2021) from chemical dominant weed control to chemical coexistent with non-chemical techniques. Developing new management tactics and implementing diverse combinations of integrated weed management present the future trend for weed control. The systematic review results showed that chemical methods had the highest weed control efficacy, while non-chemical had the lowest on average, nonetheless, all three weed control practices (chemical, non-chemical, and combined) reduced the weed biomass and density significantly compared with when no strategy was employed. Weed control challenges could be the catalyst for the development of new non-chemical and integrated weed control techniques.

## Introduction

Weeds present one of the important challenges in the nursery industry as weeds significantly affect nursery crop values by reducing their growth and salability (Amoroso et al., 2009). For instance, redroot pigweed (*Amaranthus retroflexus* L.) and large crabgrass [*Digitaria sanguinalis* (L.) Scop.] reduced Japanese holly (*Ilex crenata* Thunb. ‘Convexa’) growth by 47 and 60%, respectively, making it less or not salable (Fretz, 1972). Similarly, eclipta (*Eclipta prostrata* L.) caused a 43% reduction in Fashion azalea (*Rhododendron* × ‘*Fashion*’) growth, largely reducing its marketability (Berchielli-Robertson et al., 1990).

To control weeds, various methods have been tested, such as chemical, non-chemical, and the integrated chemical and non-chemical practices (Marble, 2015; Stewart et al., 2017). Chemical weed control primarily uses herbicides to control weeds (Altland et al., 2004) whereas non-chemical weed control utilizes different cultural practices, such as mulching, irrigation, and fertilization to reduce the weed growth (Case et al., 2005; Amoroso et al., 2009; Marble et al., 2019; Saha et al., 2019b). To reduce herbicide usage while maintaining the promising weed control results, combined chemical and non-chemical weed control have been widely developed (Altland et al., 2004; Stewart et al., 2018).

Weed control efficacy varies largely with control methods and weed species. In a field study, Diuron application at a rate of 4 kg ha^–1^ controlled 94% of weeds, which were dominated with johnsongrass (*Sorghum halepense* (L.) Pers.), green foxtail [*Setaria viridis* (L.) P. Beauv.], annual bluegrass (*Poa annua* L.), goosefoot grass (*Chenopodium album* L.), bindweed (*Convolvulus arvensis* L.), broadleaf woodsorrel (*Oxalis latifolia* Kunth.), field sow-thistle (*Sonchus arvensis* L.), bird-eye speedwell (*Veronica persica* Poir.), frost aster (*Aster pilosus* (Willd.) G.L. Nesom), black nightshade (*Solanum nigrum* L.), and scarlet pimpernel (*Anagallis arvensis* L.), however, unpunched black polyethylene resulted in 100% control of these weeds (Dalal et al., 2008). Similarly, in a container study, applying granular Broadstar 0.25 G (flumioxazin) 0.4 kg ai ha^–1^ alone controlled 92% of bittercress (*Cardamine* spp.), while combining herbicide and pine bark mini-nuggets (7.62 cm) led to 100% control efficacy (Richardson et al., 2008).

Table 1 summarizes the commonly tested weed species reported in literature from 1995 to 2021 with total 114 records. Approximately half of the studies did not specify weed species. Among the specified studies, spurge (*Euphorbia* spp.) and sedge (*Cyperus* spp.) presented to be the most tested weed species with 17 reports, followed by bittercress (*Cardamine* spp.) with 12, crabgrass (*Digitaria* spp.) with 11, and woodsorrel (*Oxalis* spp.) with 10. Bittercress (*Cardamine* spp.), spurge (*Euphorbia* spp.), and woodsorrel (*Oxalis* spp.) were mainly tested in container studies, while other weed species were reported from both field and container studies or field studies only.

**TABLE 1:** Weed species reported in literature (total 114 records) from 1995 to 2021.

Weed types	Common name	Scientific name	Number of reports	Type of study
	Forbs	*Abutilon theophrasti* Medik.	2	Field
	Common ragweed	*Ambrosia artemisiifolia* L.	2	Field
Wort and moss (5)	Mug wort	*Artemisia vulgaris* L.	1	Field
	Silver thread moss	*Bryum argenteum* Hedw.	1	Container
	Liverwort	*Marchantia polymorpha* L.	3	Container
Bittercress (12)	Wavy bittercress	*Cardamine flexuosa* With.	2	Container
	Bittercress	*Cardamine* spp.	6	Container
	Hairy bittercress	*Cardamine hirsuta* L.	4	Container
Spurge (17)	Garden spurge	*Euphorbia hirta* L.	1	Container
	Prostrate spurge	*Euphorbia humistrata* L.	8	Container
	Spotted spurge	*Euphorbia maculata* L.	8	Container
	Goosefoot	*Chenopodium* spp.	3	Field
	Horseweed	*Conyza canadensis* L.	3	Field
Sedge (17)	Sedge	*Cyperus* spp.	8	Field (7) Container
	Yellow nutsedge	*Cyperus esculentus* L.	7	Field (6) Container
	Purple nutsedge	*Cyperus rotundus* L.	2	Field
Crabgrass (11)	Southern crabgrass	*Digitaria ciliaris* Retz.	1	Container
	Smooth crabgrass	*Digitaria ischaemum* Schreb.	1	Field
	Large crabgrass	*Digitaria sanguinalis* L.	9	Container (8), Field
Barnyard grass (6)	Barnyard grass	*Echinochloa* spp.	2	Field, Container
	Awnless barnyard grass	*Echinochloa colonum* L.	2	Field
	Eclipta	*Eclipta prostrata* Roxb.	9	Container
	Goosegrass	*Eleusine indica* (L.) Gaertn.	2	Field, Container
Morning glory (3)	Ivy-leaf morning-glory	*Ipomoea hederacea* L.	1	Field
	Pitted morning-glory	*Ipomoea lacunosa* L.	1	Container
	Morning-glory	*Ipomoea* spp.	1	Field
Mallow (5)	Little mallow	*Malva parviflora* L.	3	Field
	Common mallow	*Malva sylvestris* L.	2	Field
	California burclover	*Medicago polymorpha* L.	2	Field
Woodsorrel (10)	Creeping woodsorrel	*Oxalis corniculata* L.	5	Container
	Broadleaf woodsorrel	*Oxalis latifolia* Kunth.	1	Field
	Yellow woodsorrel	*Oxalis stricta* L.	4	Container
	Annual bluegrass	*Poa annua* L.	7	Container (5), Field
	Common purslane	*Portulaca oleracea* L.	3	Field (2), Container
	Common groundsel	*Senecio vulgaris* L.	4	Container
Foxtail (5)	Giant foxtail	*Setaria faberi* Herrm.	2	Field
	Green foxtail	*Setaria viridis* L.	3	Field (2), Container
	Common chickweed	*Stellaria media* L.	8	Field, Container
	Dandelion	*Taraxacum officinale* (L.) Weber ex F.H.Wigg.	2	Field, Container
	Broadleaf weed		9	Field

Although herbicides have been largely used for nursery weed control, many challenges have emerged due to their overuse. Many tested weeds species (Table 1), such as goosegrass (*Eleusine indica* (L.) Gaertn.), annual bluegrass (*P. annua* L.), large crabgrass (*D. sanguinalis* L.), smooth crabgrass (*Digitaria ischaemum* Schreb.), and prostrate spurge (*Euphorbia humistrata* L.) have developed resistance to herbicides (Jalaludin et al., 2010; Powles and Yu, 2010; Derr et al., 2020; Boyd and Steed, 2021). In addition, it takes a long time (generally 5–10 years) to develop and register new herbicides, depending on herbicide characteristics along with other manufacturer and registration variables. The lack of discovery in new mode of reactions in recent years presented another challenge for new herbicide developments (Duke, 2012). Moreover, the overuse of herbicides, has created safety, environmental, and economic concerns due to running off, leaching, and drifting (Collins et al., 2001; Riley, 2003; Chalker-Scott, 2007).

Several weed-control reviews have been published in the past decade with focusing on one or two aspects. In Altland et al. (2003), a review focusing on different herbicides with respect to their chemical class, mode of action, and rates for field nursery was published. In Case et al. (2005) published a review on container nursery weed control practices, discussing commonly used herbicides for container nurseries, other control practices (mulching, irrigation, and combining tactics). In Marble (2015) reviewed herbicide and mulch interactions, suggesting that high mulch depths (>7 cm) resulted in a high level of weed control regardless of herbicide use. In Stewart et al. (2017), another review on container nursery and landscape weed control was published, focusing on irrigation, nutrient, and substrate management effects on the weed growth and herbicide performance. However, the evolution of a knowledge domain was rarely reported, not to mention the efficacy comparison of weed control methods, effects on weed density or biomass.

Exploring and visualizing the evolution of a knowledge domain in the past years can be achieved by detecting remarkable articles in citation and co-citation networks from each time interval, and major changes between adjacent time series in a panoramic view (Chen, 2004). Chen (2004) proposed using the software CiteSpace to visualize salient nodes in merged networks from a specific knowledge area, and described three types of nodes: (a) landmark node with a large radius, representing the most highly cited documents; (b) hub node with a large degree, indicating widely cited papers with significantly intellectual contributions; and (c) pivot nodes with two networks exclusively connected by few lines, presenting common knowledge shared by different knowledge focus areas.

Systematic reviews and meta-analyses have been widely used for quantitative research reviews as they rely on the quantitative information and allow for the testing of hypotheses that cannot be satisfactorily answered by a single study (Shrestha et al., 2016; Osipitan et al., 2018). Systematic reviews reduce bias by appraising and synthesizing the surveyed studies based on a set of criteria to answer a specific question (Phan et al., 2015). Meta-analysis is a statistical technique summarizing the data extracted through a systematic review into a single quantitative estimate of effect sizes (Haddaway et al., 2015).

As such, this research aimed at analyzing how the evolution of knowledge domains in nursery weed control changed over time and visualizing the trends and linkages of the main scientific research areas. A systematic analysis was also conducted to evaluate the different weed control methods efficacy. Additionally, a meta-analysis was carried out to evaluate the relative impact of different weed control methods on weed biomass and density.

## Materials and Methods

### Literature Search and Data Collection

**FIGURE 1:**
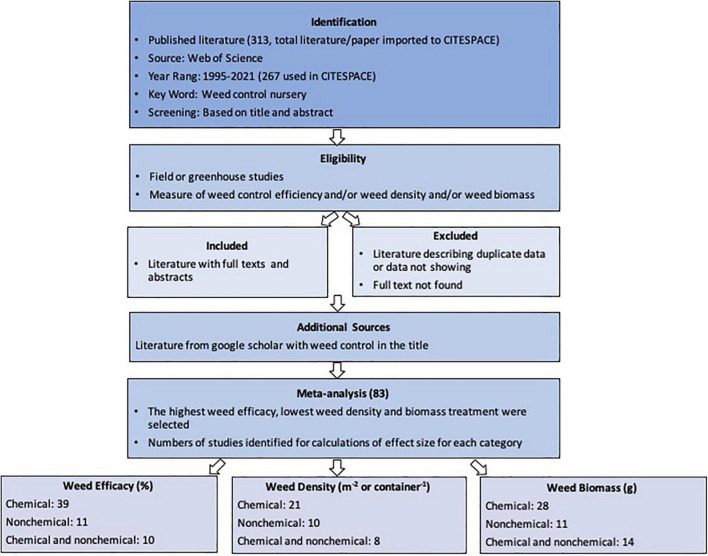
Flow diagram showing the study selection procedure.

**FIGURE 2:**
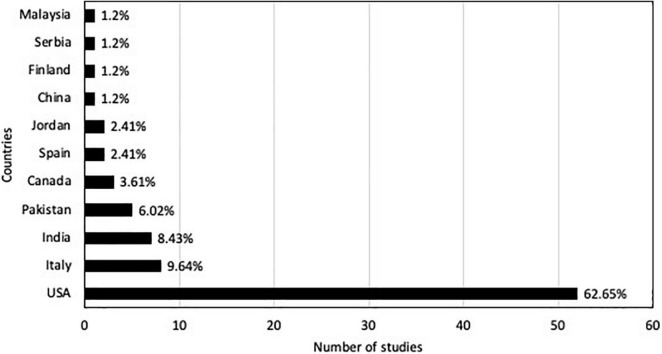
Countries of the studies reported in the articles used for the meta-analysis.

The primary literature search was performed by using the Web of Science database using the term “nursery weed control” on August 31, 2021. No language restriction was applied, and years of publication were from 1995 to 2021, resulting in 267 records in total. Additional relevant peer-reviewed publications were searched using “Google scholar™” with the keyword “weed control.” Duplicate references were removed (Figure 1). Studies with reports on weed efficacy, weed density, and/or weed biomass were selected for the meta-analysis to estimate the common truth for the different control methods effects, categorized in chemical, non-chemical, and integrated chemical and non-chemical practices. The meta-analysis included 83 publications in total with studies from 11 countries (Figure 2). Some of the reports were selected for all categories because they reported all the parameters (weed efficacy, weed density, and weed biomass). Biological weed control was not included or discussed in this study.

### Criteria for Paper and Data Selection

In CiteSpace, two criteria were used for the paper selection: g-index and top 50 usages since 2013. References with citations numbers more than 2 were displayed. For systematic and meta-analysis reviews, the best weed control methods with the highest weed efficacy, and/or the lowest weed biomass and density were selected in each study. Both field studies and container studies were included, with weed density units for field and container studies being weed number per square meter and weed number per container, respectively. Units for biomass were unified to gram (g), both fresh weight and dry weight were included. All three parameters (weed efficacy, weed biomass, and weed density) were compared with non-weed control treatments.

### Data Extraction and Analysis

Software GetData Graph Digitizer (version 2.26) was used to extract data from figures. When SEs were not presented or could not be calculated, we assumed a SE of 10% of the mean (Rose et al., 2014). All the citations (full report) were analyzed with the software CiteSpace (current version 5.8. R2). A meta-analysis was conducted by using the *meta* package in R Studio (Version 1.3.1093). Fixed and random-effects models were both used in the meta-analysis to provide more unbiased information: fixed effects assume all studies with the size of their effect come from a single population (with a single source of variance), studies with greater precision (large study number and small SE) have a higher weight to affect overall effects; random effects add another variance in addition to the fixed effects to count for the variability of the true effect size, in this case, small studies play more important roles in overall effects (Harrer et al., 2021). A meta-analysis was conducted for weed density and biomass, respectively. Since the weed efficacy for the control was 0, we analyzed the weed control efficacy with boxplots using the R Studio.

## Results

### Research Trends

**FIGURE 3:**
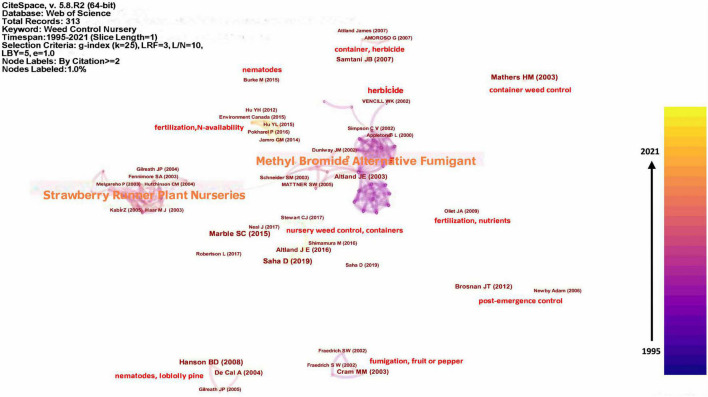
CiteSapce generated knowledge networks from 1995 to 2021. The red texts are keywords derived from the clusters or the literature next to them.

**FIGURE 4:**
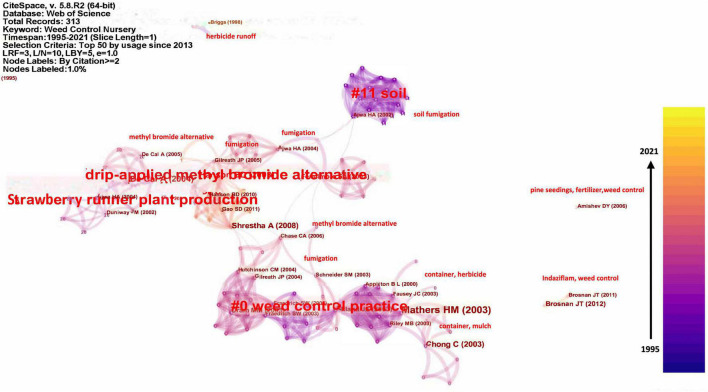
CiteSapce generated knowledge networks on the top 50 pieces of literature used since 2013. The small red texts are keywords derived from the clusters or the literature next to them.

The network was divided into different clusters based on the influential articles and their citations (Figures 3, 4), with the more intense crosslink (where nodes gather together) labeled by the most frequently used title terms from the literature (Chen, 2006). In Figure 3, “methyl bromide alternative fumigant” was the hub node connecting the common knowledge or the close research areas, meaning the focus on weed control in nurseries was the methyl bromide alternative fumigants while the pivot nodes connecting other topics (e.g., herbicide) and strawberry runner plant nurseries. Other pivot nodes at different time slices were distributed at different places and were not connected to the hub nodes as indicated by the scattered/non-connected pivot nodes.

Based on the pub nodes and pivot nodes, two clusters were divided and labeled by index terms from their citers. In the first decade (1995–2005), chemical, primarily methyl bromide alternative fumigants, along with the strawberry runner plant nurseries, were the main focuses in the nursery weed control (Duniway, 2002; Cal et al., 2004; De Cal et al., 2005; Hanson et al., 2010). At this time, weeds and pathogens (mainly nematodes) were primarily controlled using methyl bromide alternatives (Schneider et al., 2003; Gilreath et al., 2004; De Cal et al., 2005; Hanson et al., 2010). Besides the two major clusters, other literature focused on herbicides, container weed control, and/or nematodes but not necessarily closely related to the hub nodes (Vencill, 2002; Mathers, 2003; Gilreath et al., 2005).

In the following years (2006–2021), the research focus switched more toward nursery/container weed control and weed practices. New weed control methods, such as chemicals, primarily herbicides, as well as non-chemical, primarily mulching, and fertilization have gained more attention in recent years. In Figure 3, the separated pivot nodes at different time slices show that the studies on nursery/container weed control, fertilizers, and herbicides are not well connected. The literature was widely distributed based on various topics, such as weed control methods (herbicide, fertilization, and mulch) as well as crops (pine trees, peaches, and ornamentals), indicated by the scattered pivot nodes (Hanson and Schneider, 2008; Brosnan et al., 2012; Altland et al., 2016; Stewart et al., 2018; Massa et al., 2019; Saha et al., 2019a).

Since research interests have been largely switched from the first decade (1995–2005) to the following years (2006–2021), we derived a literature usage on “nursery weed control” since 2013 (Figure 4). In Figure 4, “weed control practice” became the hub node, meaning that the research interests have switched to different weed control practices, such as herbicide, mulching, fertilization, combinations of herbicide, and other practices since 2013. The pivot nodes connecting different time slices showed that the integration of studies on weed control practices, methyl bromide alternatives, strawberry runner plant productions, and soil fumigations were well connected (Chen, 2006).

Based on the pub nodes and pivot nodes, four clusters were divided and labeled by index terms from their citers, such as weed control practices, strawberry runner plant production, drip-applied methyl bromide alternative, and soil fumigation. The weed control practice literature group was mainly cited by container studies investigating mulching, fertilization, herbicides, or chemical and non-chemical combinations. However, the other three groups were cited by field studies in the first decade and both field and container studies in the following years.

### Different Weed Control Methods on Weed Control Efficacy

**FIGURE 5:**
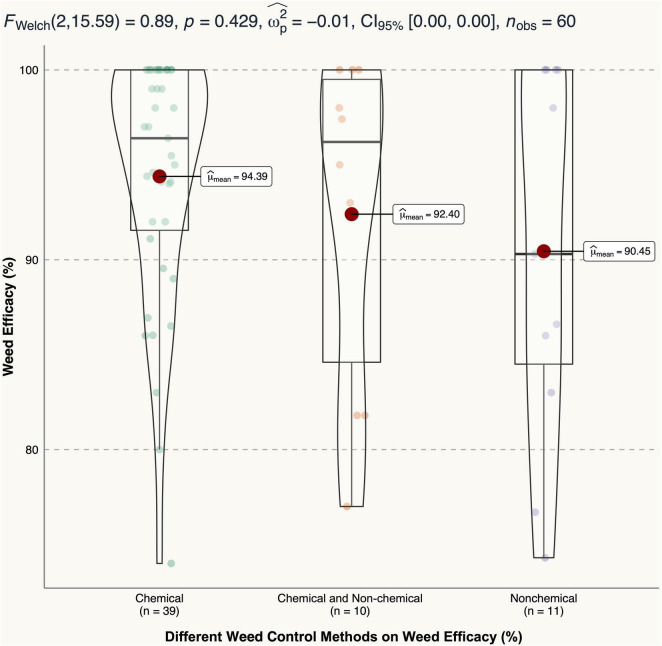
Different weed control methods (chemical, chemical and non-chemical combined, and non-chemical) effects on weed control efficacy.

The highest weed control efficacy was 100% and all three weed control methods can reach 100% efficacy depending on weed species and practices (Figure 5). The lowest weed control efficacy for chemical, non-chemical, and the combined were 74, 74.3, and 81.8%, respectively. On average, chemical methods had the highest weed control efficacy mean (94.39%), and non-chemical had the lowest mean (90.45%) among the three, although no significant difference was detected (*p* = 0.429).

### Chemical Effects on Weed Biomass and Density

**FIGURE 6:**
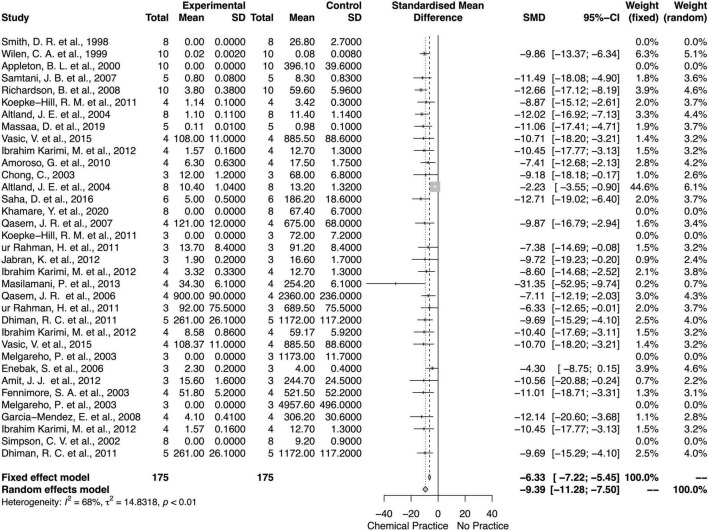
Meta-analysis for the chemical weed control method on weed biomass.

**FIGURE 7:**
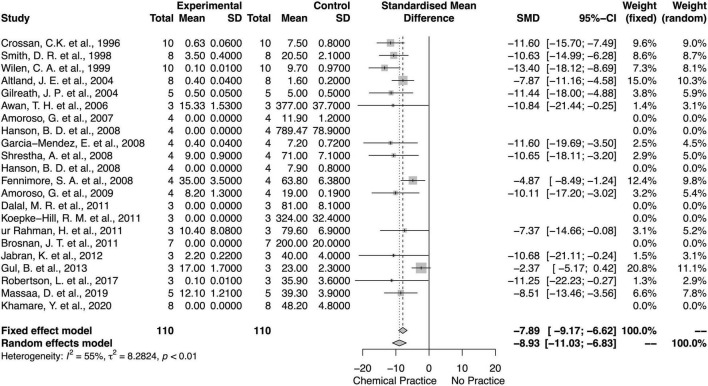
Meta-analysis for the chemical weed control method on weed density.

For both fixed and random models, the application of chemical weed control method decreased weed biomass and density (Figures 6, 7). Diamonds in the meta-analysis showed the overall effects (or standard mean differences, SMD) and its 95% *CI* of comparing chemical application with no chemical application. There was an overall mean difference between with or without chemical application effects on weed biomass (SMD = −9.39; 95% *CI* = −11.28, −7.5; *p* < 0.01, random model) and weed density (SMD = −8.9; 95% *CI* = −10.95, −6.85; *p* < 0.01, random model). Overall, there was moderate heterogeneity (*I*^2^ = 68 and 55%, respectively) among studies on chemical effects on the weed biomass and density (Mikolajewicz and Komarova, 2019).

### Non-chemical Effects on Weed Biomass and Density

**FIGURE 8:**
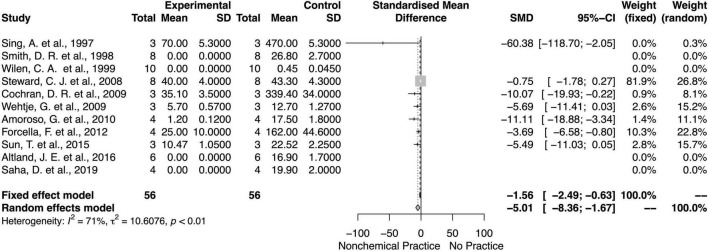
Meta-analysis for the non-chemical weed control method on weed biomass.

**FIGURE 9:**
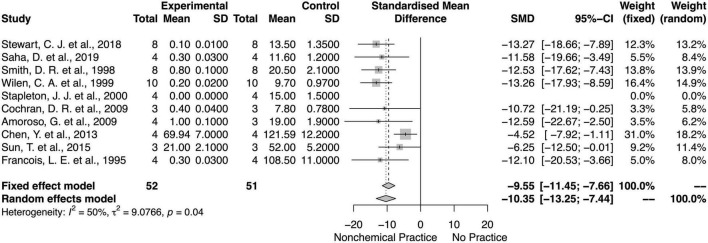
Meta-analysis for the non-chemical weed control method on weed density.

Similarly, non-chemical weed control methods decreased the weed biomass and density (Figures 8, 9). There was an overall mean difference between with or without non-chemical method effects on the weed biomass (SMD = −4.84; 95% *CI* = −7.97, −1.91; *p* < 0.01, random model) and weed density (SMD = −10.33; 95% *CI* = −13.19, −7.47; *p* = 0.04, random model). Overall, there was moderate heterogeneity (*I*^2^ = 71 and 50%) among studies on non-chemical effects on the weed biomass and density, respectively.

### Chemical and Non-chemical Effects on Weed Biomass and Density

**FIGURE 10:**
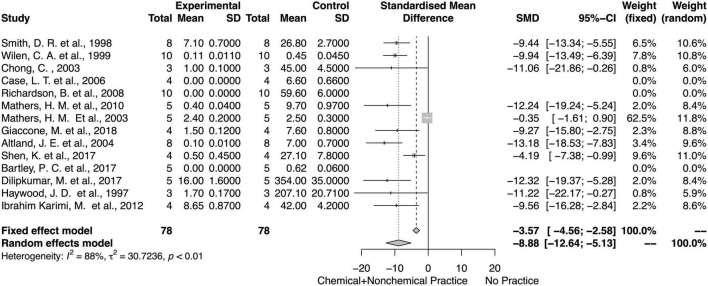
Meta-analysis for the chemical and non-chemical combined weed control method on weed biomass.

**FIGURE 11:**
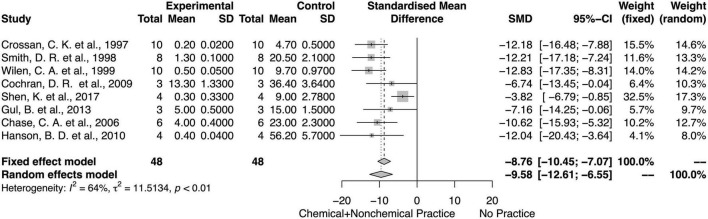
Meta-analysis for the chemical and non-chemical combined weed control method on weed density.

For both fixed and random models, integrated chemical and non-chemical weed control methods decreased the weed biomass and density (Figures 10, 11). There was an overall mean difference between with or without integrated chemical and non-chemical method effects on weed biomass (SMD = − 8.57; 95% *CI* = −11.49, −5.65; *p* < 0.01, random model) and weed density (SMD = −9.54; 95% *CI* = −12.34, −6.74; *p* < 0.01, random model). The heterogeneity among studies on integrated chemical and non-chemical effects on the weed biomass was high (*I*^2^ = 88%) and moderate on weed density (*I*^2^ = 64%, respectively).

## Discussion

### Meta-Analysis Heterogeneity

Heterogeneity was defined as the proportion of total variance between studies, indicating the inconsistency between studies (Mikolajewicz and Komarova, 2019). The value of *I*^2^ ranges from 0 to 100% and does not depend on the number of comparisons in the meta-analysis. The values of *I*^2^ at 25, 50, and 75% reflect low, moderate, and high heterogeneity, respectively (Higgins and Thompson, 2002). The decision to use a fixed-effects or random-effects model based on these statistics is subjective; however, we would consider using a random-effects model on *I*^2^ values greater than 50% (Osipitan et al., 2018). There was high heterogeneity among studies for most measurements of weed biomass and density, with the highest *I*^2^ being 88%, found in chemical and non-chemical combined effects on weed biomass studies (Figure 10). However, among the studies for the effects of non-chemical weed control method on weed density, 50% of the primary studies were at variance. The moderate heterogeneity was acceptable and might be because some research studies (e.g., container studies) were conducted with more replications and less variance than others (e.g., field studies).

The main sources of heterogeneity in this study could because of the differences in weed species and control practices: weed species, such as spotted spurge (*Euphorbia maculata* L.), oxalis (*Oxalis corniculate* L.), northern willowherb (*Epilobium ciliatum* Raf.), and common groundsel (*Senecio vulgaris* L.); practices, such as use rice hull mulch, steaming, fertilizer placements, and different chemicals (isoxaben, trifluralin, indaziflam, prodiamine, methyl bromide, and chloropicrin). To address differences among the primary studies, a random-effects model was included and used for the meta-analysis. The random model recognized the variance among studies and summarized the effect sizes as weighted means based on these differences (Osipitan et al., 2018; Mikolajewicz and Komarova, 2019).

### Research Trend in Nursery Weed Control

The research interests in nursery weed control have switched dramatically in the past 2–3 decades (1995–2021) from chemical dominant weed control to chemical coexist with other techniques. In the first decade, chemical weed control obtained the major research focus, specifically methyl bromide alternatives, due to the phase-out of methyl bromide (Keddy et al., 1996; Duniway, 2002; Cal et al., 2004; Enebak et al., 2006; Fennimore et al., 2008; Garcia-Mendez et al., 2008). Among the 267 pieces of literature, 27 focused on methyl bromide alternatives. In the meantime, non-chemical weed control techniques, specifically disks, occupied a small portion (2 out of 267) of the research interests (Chong, 2003; Knox et al., 2012). As a result of methyl bromide phase-out, herbicide become a research hot spot in the nursery weed control (78 out of 267). Since 2013, due to the increasing environmental concerns, research switched to weed control practices (Figure 3, 128 out of 267), specifically on non-chemical as well as chemical and non-chemical integrated. Among them, mulch started to draw attention of researchers and became the widest tested method (29 out of 267).

Not only research interests have changed dramatically, but also chemical products used in weed control. At methyl bromide phase-out times, the common tested chemicals included chloropicrin, metam sodium, 1,3-dichloropropene, iodomethane, dazomet, anhydrous ammonia, and their combinations with different formulations (Reynolds et al., 2002; Schneider et al., 2003; Gilreath et al., 2004, 2005; Fennimore et al., 2008; Shrestha et al., 2008; Schneider and Hanson, 2009; Hanson et al., 2010). Some of those chemicals, such as iodomethane reached a 100% weed control efficacy (Fennimore et al., 2008). Later, herbicides, such as oryzalin, glyphosate, flumioxazin, oxyfluorfen, pendimethalin, isoxaben, trifluralin, and their combinations become more widely tested, other herbicides that have both preemergence and postemergence effects, such as quinoclamine was also tested in different studies (Porter, 1996; Willoughby et al., 2003; Altland et al., 2004, 2007, 2008, 2011; Awan et al., 2006; Judge and Neal, 2006; Qasem, 2006, 2007; Richardson and Zandstra, 2009; Wehtje et al., 2010a,b, 2012; Rahman et al., 2011; Abit and Hanson, 2013; Ramalingam et al., 2013; Vasic et al., 2015; Marble et al., 2016b). Most of the herbicides, such as oxadiazon, flumioxazin, and prodiamine, presented satisfactory weed control performances (Judge and Neal, 2006; Amoroso et al., 2009; Wehtje et al., 2010a).

Similarly, non-chemical weed control experienced significant change. In the early years of the evaluation period, different disks, such as Geo-disk, non-woven polypropylene fabric, plastic lids, and polyethylene sleeves were tested (Case et al., 2005; Lanthier et al., 2006; Knox et al., 2012; Sun et al., 2015). However, due to the poor weed control performance, most weed disks introduced during the past 15 years were no longer being used or sold (Chong, 2003). Later, mulches stood out among other non-chemical practices and became a research hot spot due to their easy availability and low prices (Chalker-Scott, 2007). The widely tested mulches, such as pine bark, rice hull, Douglas fir bark, coconut coir, newspaper pellets, and waste paper (Pellett and Heleba, 1995; Penny and Neal, 2003; Amoroso et al., 2009; Mathers and Case, 2010; Chen et al., 2013; Marble, 2015; Bartley et al., 2017; Burrows, 2017; Masilamany et al., 2017; Marble et al., 2019; Massa et al., 2019). Adding mulches (2.54–7.62 cm) can reach satisfactory weed control results depending on mulch types and weed species (Richardson et al., 2008; Cochran et al., 2009; Altland et al., 2016; Massa et al., 2019; Särkkä and Tahvonen, 2020). In addition, innovative non-chemical weed control methods, such as fertilizer placement, irrigation, flaming, steaming, oil palm, real-time robotics have been proposed and tested (Rainbolt et al., 2013; Frasconi et al., 2014; Masilamany et al., 2017; Pokharel et al., 2017; Stewart et al., 2017, 2018; Saha et al., 2019b; Raja et al., 2020).

Similarly, the chemical and non-chemical integrated weed control practices evolved from herbicide combined with mechanical to herbicide combined with mulch. In the first decades, chemical and disks were the two main ways for weed control, only a few studies tested chemical and non-chemical (mechanical or hand weeding) combined (Zheljazkov et al., 1996). In recent years, researchers integrated mulches and herbicides for weed control, aiming to reduce herbicide usage (or increase weed control performance) while maintaining the sufficient weed control (Somireddy, 2011; Masilamany et al., 2017; Shen and Zheng, 2017; Giaccone et al., 2018; Anthony and Witcher, 2020). Case and Mathers (2006) showed that applying SureGuard (flumioxazin) at half manufacturer label rate (0.19 kg ai ha^–1^) to hardwood or rice hull significantly reduced spurge (*Euphorbia maculata* L.), annual bluegrass (*P. annua* L.), and common chickweed (*Stellaria media* L.) biomass. In addition, Richardson et al. (2008) showed that applying pine bark mini-nugget mulch (3.8 cm) with Broadstar (flumioxazin) herbicide increased weed control efficacy comparing with the non-treated control, herbicide alone, or mulch alone, achieved a 100% weed control efficacy for both bittercress (*Cardamine* spp.) and oxalis (*Oxalis stricta* L.). Other combinations of herbicides and other non-chemical practices have also been tested (Wehtje et al., 2009).

With the rising environmental concerns and potential restrictions, herbicide alternatives are becoming more important (Chong, 2003). Weed science is extending from a discipline emphasizing herbicides toward a multidiscipline integrating several weed control methods (Hamill et al., 2004). Developing new management tactics, and implementing diverse combinations of integrated weed management present the future trend for weed control (Harker and O’Donovan, 2013; Stewart et al., 2017).

### Weed Control Efficacy in Nursery Weed Control

For the same weed species, different methods led to the varied weed control. For a mix of annual bluegrass (*P. annua* L.), common groundsel (*Senecio vulgaris* L.), and shepherd’s purse (*Capsella bursa-pastoris* L.), applying Diuron 80DF (diuron) alone at 1.12 kg ai ha^–1^ resulted in 86% control; however, treating weeds with diuron at 1.12 kg ai ha^–1^ and rice hull (0.51 cm) reached to 98% control; leaf pellet application controlled 50% weeds (Samtani et al., 2007). Similarly, in a container study, applying Rout (oxytluorfen + oryzalin) at 3.3 kg ai ha^–1^ for spotted spurge (*Euphorbia maculata* L.) resulted in 3.5 weed density (number per container); treating weeds with fabric disk plus Spin Out (PGR) led to 1.3; yet using recycled paper pellets reduced weed density to 0.8 (Smith et al., 1998).

The target weed species had a significant impact on weed control efficacy. Granular flumioxazin at 0.41 kg ai ha^–1^ controlled 84% doveweed (*Murdannia nudiflora* L.) but only 22% crabgrass (*D. sanguinalis* (L.) Scop.). Pendimethalin + dimethanamid-P (2.24 + 1.68 kg ai ha^–1^) resulted in 100% weed control on doveweed (*Murdannia nudiflora* L.) and large crabgrass (*D. sanguinalis* (L.) Scop.), but only 19% on eclipta (*Eclipta prostrata* L.) (Saha et al., 2016). In a container study, applying pine bark mini nuggets at 2.54 cm controlled 87% spotted spurge (*Euphorbia maculata* L.) and 89% eclipta (*Eclipta alba* L.) (Cochran et al., 2009). In a field study, hand weeding followed by 0.0024 kg ai ha^–1^ imazethapyr-treated oil palm frond mulch (3.4 t ha^–1^) controlled 94.5% Ganges primrose [*Asystasia gangetica* (L.) T. Anderson], 94.0% of junglerice [*Echinochloa colona* (L.) Link], 96.4% of mixed switchgrass species (*Panicum* spp.), 99.0% mile-a-minute (*Mikania micrantha* Kunth), and 96.8% of gale-of-the-wind (*Phyllanthus amarus* L.) 3 months after treatment in the coconut plantations (Masilamany et al., 2017).

It is commonly known that weed control is determined by both methods and species. How do we select the right practice for weed control? In general, chemical methods had better weed control efficacy compared with other control methods (Figure 5). However, weed density and biomass results varied as practices changed. Applying chemical products reduced weed density the most (by 94.6%), followed by non-chemical (85.7%), and integrated (80.8%). However, integrated methods reduced weed biomass the most (by 95.0%), followed by non-chemical (86.4%), and chemical (82.6%).

Unlike agronomic crops, most nursery crops are sold and marketed based on the aesthetic value, and consumers demand weed-free pots (Simpson et al., 2002). Thus, we removed all the field studies and got the average weed control results for each method. The non-chemical method reduced weed density the most (by 97.1%), followed by chemical (by 87.1%), and integrated (by 80.6%). In terms of weed biomass, these three methods presented similar effectiveness for weed control, although the chemical had the most control effects (by 95.3%), followed by integrated (by 93.2%) and non-chemical (by 90.3%).

As such, in container studies, non-chemical methods can reach the best weed control performance as comparing with chemical or integrated methods. The average number of weed per container was 0.4 for non-chemical method and was 2.8 for chemical. Although only one non-chemical study showed 100% control for weed density (Stapleton et al., 2002), other non-chemical studies had nearly 0 weed density (0.2–0.4) (Wilen et al., 1999; Cochran et al., 2009; Saha et al., 2019a). For chemicals, however, although some studies had close to 0 weed density (e.g., 0.1 per container) (Robertson and Derr, 2017), many other studies had larger weed density (e.g., 3.5–12.1) (Smith et al., 1998; Massa et al., 2019). For both weed density and biomass, the integrated method presented a medium effect on weed control, similar to the weed efficacy results (Figure 5). The reason might be because some studies showed relatively high control performance for weed density (0.2–0.3) and biomass (0 g) (Crossan et al., 1997; Bartley et al., 2017; Shen and Zheng, 2017).

### Challenges and Opportunities in Nursery Weed Control

Since 2005 following the phase-out of methyl bromide, herbicides have become the dominant chemical weed control method in nurseries because they are highly effective on most weeds. Herbicides account for 60% of the pesticides used worldwide, and most large-scale crop production systems rely extensively on synthetic herbicides to manage weeds (Dayan, 2019). About 2.2 Mt of herbicide was used worldwide in 2019 with the United States contributing 0.26 Mt of herbicide usage, ranking the second largest herbicide usage country following China (FAOSTAT, 2021).

However, the wide use of herbicides has brought many challenges, with the first being herbicide-resistant weeds. Weeds can adapt to new herbicides and exhibit herbicide resistance in a short time (Burrows, 2017). Since 1985, reports of herbicide-resistant weeds increased from less than 100 cases in 1985 to nearly 500 cases globally in 2019. Over 23 weed species from 20 countries have been confirmed glyphosate resistance, and triazine resistant weed species have been largely confirmed in recent years (Powles and Yu, 2010; Derr et al., 2020).

The wide use of herbicides has caused environmental concerns and economic losses due to its leaching, runoff, and spray-drift (Briggs et al., 2002; Riley, 2003; Case and Mathers, 2006). For instance, up to 86% of a granular applied herbicide can be lost by misapplication and non-target loss, depending on the pot spacing and species (Gilliam et al., 1992). In addition, to maintain an acceptable weed control, nurseries often conduct frequent reapplications, leading to more runoff and leaching, causing environmental concerns (Horowitz and Elmore, 1991; Crossan et al., 1996, 1997; Bana et al., 2020). Moreover, herbicides drift from nearby farm fields could lead to crop damaged greater than 50%, causing huge economic losses depending on the farm size and plant species (Derr et al., 2020).

The costly and time-consuming herbicides development and regulation present another big challenge, especially in the ornamental industry (Altland et al., 2004; Duke, 2012). In the last 20 years, only a few new modes of action (e.g., indaziflam cellulose biosynthesis inhibitors) have been registered for use in nurseries (Duke, 2012; Brabham et al., 2014). Additionally, for the last 10 years, only very few new herbicides have become available to the landscape sector, with fewer postemergence herbicides (Case and Mathers, 2006; Marble, 2015).

However, on the bright side, herbicide weed control challenges could be the catalyst for the development of new non-chemical and integrated weed control techniques, creating new opportunities (Walsh et al., 2013). For instance, new mulch materials with satisfactory weed control performance could be explored and developed (Marble, 2015). Exploring new substrates and testing how different substrates can affect the weed growth can be another research direction. More research on how properties of mulches, such as particle size and feedstock influence the nursery weed control need to be conducted. Studies on how different mulch–herbicide combinations affect the weed growth need to be examined, especially with postemergence herbicides (Marble, 2015; Marble et al., 2016a). Furthermore, how different mulch materials affect herbicide leaching and runoff after application need to be determined (Marble, 2015). Moreover, understanding the mechanisms of how mulch controls weed will help the horticulture industry (Saha et al., 2018).

## Conclusion

In this review, we provided a visualization and systematic understanding of research trend on nursery weed control. The results showed that interests in nursery weed control have switched dramatically in the last 2–3 decades (1995–2021) from chemical dominant weed control to chemical coexist with non-chemical techniques. The meta-analysis results indicated that all three weed control practices (chemical, non-chemical, and combined) reduced weed biomass and density significantly. With the rising environmental concerns and potential restrictions, developing new management tactics, implementing diverse combinations of integrated weed management present the future trend for weed control.

## Data Availability Statement

The original contributions presented in the study are included in the article/supplementary material, further inquiries can be directed to the corresponding author.

## Author Contributions

PY conducted the literature searching, collected and analyzed the data, and wrote the manuscript with the assistance of SCM. Both authors contributed to the article and approved the submitted version.

## Conflict of Interest

The authors declare that the research was conducted in the absence of any commercial or financial relationships that could be construed as a potential conflict of interest.

## Publisher’s Note

All claims expressed in this article are solely those of the authors and do not necessarily represent those of their affiliated organizations, or those of the publisher, the editors and the reviewers. Any product that may be evaluated in this article, or claim that may be made by its manufacturer, is not guaranteed or endorsed by the publisher.
